# Social cognitive determinants of ecstasy use to target in evidence-based interventions: a meta-analytical review

**DOI:** 10.1111/j.1360-0443.2007.02041.x

**Published:** 2008-01

**Authors:** Gjalt-Jorn Y Peters, Gerjo Kok, Charles Abraham

**Affiliations:** 1 Department of Work and Social Psychology, Faculty of Psychology, Maastricht University the Netherlands, UK; 2 School of Social Sciences, University of Sussex UK

**Keywords:** Determinants, drugs, ecstasy, expectancies, review, theory of planned behaviour

## Abstract

**Aims:**

The health hazards and prevalence of ecstasy use have been documented in two decades of research, but no review reporting on potentially modifiable antecedents of use is available. The aim of this study was to integrate systematically research identifying cognitive correlates of ecstasy use. Such research has the potential to identify targets for evidence-based interventions designed to discourage use.

**Methods:**

The databases PsycINFO and MedLine were searched, inclusion criteria applied to resulting hits, and descendency and ancestry approaches applied to the selected publications. Reported associations between cognitive determinants, including intention to use and ecstasy use measures, were synthesized by calculating a weighted mean effect size, *r*.

**Results:**

The pattern of associations lent support both to the theory of planned behaviour (TPB) and the expectancy approach as descriptions of potentially useful determinants. Attitudes were associated most strongly with intention and use, followed by subjective norm and perceived behavioural control.

**Conclusions:**

Consideration of the strength of associations and the potential modifiability of identified cognitions suggests that evidence-based interventions to discourage ecstasy use should target negative expectancies, perceived behavioural control and anticipated regret, and consider tailoring perceived behavioural control elements.

## INTRODUCTION

Ecstasy use is potentially damaging to health [1–3] yet prevalent [4,5]. Legislative changes have not been effective in discouraging ecstasy use, and the development of theory-based behavioural interventions is warranted because these have been successful in generating behaviour change in other areas [6,7].

Behaviour change interventions are more likely to be effective if they target modifiable antecedents of the target behaviour. For example, if expected positive outcomes of a target behaviour differentiate between those who do and do not engage in that behaviour, it is prudent to target outcome expectancies in behaviour change interventions [8]. Which potentially modifiable cognitive antecedents of ecstasy use should interventions target? Unfortunately, although ecstasy was synthesized in 1912 [9], and ecstasy use has been studied for 20 years (e.g. [10,11]), there is no systematic review of this research identifying potentially modifiable cognitive antecedents of use. We aimed to summarize research to date, synthesizing quantitatively all published, quantitative studies of psychological determinants of ecstasy use among young people living in western society.

## METHOD

The search strategy comprised three iterative steps. First, the databases PsycINFO, MedLine and ERIC (Education Resources Information Center) were searched using several combinations of keywords (see Appendix I). The results of the final query were then scanned manually for relevant entries by examining the paper titles and abstracts (see Appendix II). Four inclusion criteria were used. Firstly, a study should investigate the target population of young recreative ecstasy users in western society, as factors influencing behaviour can be population-specific [12–14]. Secondly, a study should measure one or more potentially modifiable determinants of ecstasy use-related behaviour; that is, an antecedent that could be influenced potentially by health promotional interventions (excluding for example demographics, personality, etc.); for a list, see chapter 7 of Bartholomew *et al*. [8]. Thirdly, the study should measure either actual behaviour or intention. Finally, it should assess quantitatively the relationship between determinants and behaviour or intention. Publications selected by this process were examined in detail. Second, reference lists of these papers were scanned for relevant publications (the ancestry approach). Third, texts citing the relevant papers were located using the Web of Science database (the descendancy approach).

Potentially modifiable determinants were extracted from the studies using a recommended published list [8], on the basis of which two authors selected determinants from each paper and perfect agreement was observed. Associations between determinants and behaviour or intention across studies were integrated by converting all statistics to the correlation coefficient *r*. These coefficients were then transformed to Fisher's *Z* and weighed by sample size −3 (cf. [15]). The mean Fisher's *Z* was then transformed back to the correlation coefficient *r*+. When a study tested a variable several times (e.g. frequency of use and intensity of use), the resulting effect sizes were averaged before being included in the calculations.

## RESULTS

The search yielded 367 hits, from which 15 publications were included (see Appendix II). Many excluded studies had a biological focus (e.g. [3]), examined determinants not feasibly changed by health promoting interventions (e.g. [16]) or used qualitative methods (e.g. [17]). All included publications studied the behaviour ‘using ecstasy’ (or the intention to use); none examined determinants of trying out ecstasy, ceasing use, changing use patterns or applying harm reduction practices. Of these 15 publications, six were discarded after thorough examination, as they were then discovered to yield no quantitative information on the relevance of potentially modifiable determinants of behaviour in the target population ([18–23]; see also Appendix II). Application of the ancestry approach yielded no additional publications (365 citations scanned), but the descendancy approach yielded one additional publication (of 85 unique citing publications; [24]). Of the final set of 10 publications [24–33], one publication described two studies ([26]; 26a and 26b refer to studies 1 and 2, respectively). Table 1 describes the 11 included studies, listing the sample details, the extracted potentially modifiable antecedents and how they were measured in the original studies.

**Table 1:** Quantitative studies into the determinants of using ecstasy and measures used.

No.	Sample details	n	Age	% ♀	Relevant variables	I	R
[24]	UK, before July 2002 Polydrug users	364	19	44%	Negative mood function scale	3	5
					Social function scale	5	5
					Negative effects	4	5
					Other functions (9 functions)	1	5
					Extent of peer use	1	4
					Partner/best friend use	1	2
					Intensity of use	1	–
[25]	UK, before March 1998 Alcohol and drug users	100	19	45%	Mood function scale	3	5
					Social/contextual function scale	5	5
					Negative effects/events scale	3	5
					Extent of peer use	1	5
					Intention	1	7
					Intensity of use	1	–
					Frequency of use	1	–
[26a]	UK, March 1992 Students	186	19–25	58%	Attitudes	6	7
					Subjective norms	1	7
					Perceived behavioural control	6	7
					Behavioural beliefs (17)	2	19
					Normative beliefs (5)	2	19
					Control beliefs (8)	2	19
					Intention to use ecstasy	4	7
[26b]*	UK, mid-1996 Club members	t1: 203 t2: 123	23	41%	Attitudes	8	7
					Normative influences	13	7
					Perceived behavioural control	1	7
					Self-efficacy	1	7
					Behavioural beliefs	13	7
					Control beliefs	6	7
					Intention	3	7
					Behaviour (longitudinal)	4	–
[27]	Netherlands, 2000, 2001 Party attendants	844	22	33%	Negative outcome expectancies	11	2
					Enhancement outcome expectancies	3	2
					Euphoria outcome expectancies	4	2
					Sex outcome expectancies	4	2
					Dancing outcome expectancies	3	2
					Insight outcome expectancies	4	2
					Communication outcome expectancies	4	2
					Whether ecstasy was currently used	1	2
[28]*	UK t1: October 1994–1995 t2: May1995–1996 Students	t1: 461 t2: 136	19–22 19–22	55% 65%	Attitude	2	7
					Injunctive norms	6	7
					Perceived behavioural control	8	7
					Descriptive norms	2	6
					Moral norm	1	7
					Intention	1	8
					Behaviour (longitudinal)	1	8
[29]	UK, before 2003 College students	657	19	55%	Frequency of past use	1	7
					Intentions to use	1	9
					Normative influence (friends’ use)	1	6
					Beliefs about ecstasy use (7 beliefs)	1	5
[30]*	UK	t1: 84 t2: 32	20	74%	Attitude	10	8
					Subjective norm	2	8
					Perceived behavioural control over obtaining ecstasy	3	8
					Perceived behavioural control over taking ecstasy	4	8
					Intention	5	8
					Habit	2	8
					Specific attitudinal beliefs (13 beliefs)	1	5
					Behaviour (longitudinal)	1	2
[31]	the Netherlands, 2001–2002	490	22	34%	Energy motives	4	5
					Euphoria motives	3	5
					Self-insight motives	2	5
					Sociability/flirtatiousness motives	8	5
					Sexiness motives	4	5
					Coping motives	3	5
					Conformism motives	4	5
					Perceived positive effects	24	2
					Perceived negative effects	11	2
					Perceived friends’ use	1	5
					Frequency of ecstasy use	1	5
[32]	UK	200	21	66%	Attitude	5	?
					Subjective norm	5	5
					Perceived behavioural control over obtaining ecstasy	3	7
					Perceived behavioural control over taking ecstasy	11	?
					Intention	6	7
					Habit	2	7
[33]	USA Club rave attendees	70	20	47%	Risk associated with using ecstasy once or twice	1	4
					Risk associated with using ecstasy regularly	1	4
					Harmful short-term physical effects	1	4
					Harmful long-term physical effects	1	4
					Harmful short-term psychological effects	1	4
					Harmful long-term psychological effects	1	4
					Positive physical effects	1	4
					Positive psychological effects	1	4
					Ecstasy use within the past 12 months	1	2

No. = number in reference list, I = number of items used to measure variable, R = number of scale points on response scale of each item.

* Longitudinal design.

All 11 studies can be viewed as tests of two theoretical frameworks which are applied frequently in drug use research [34]. Six studies [26a,26b,^28^–^30^,^32^] tested the theory of planned behaviour (TPB; [35]). The TPB proposes that the most proximal cognitive determinant of behaviour is intention which, in turn, is predicted by attitude (i.e. evaluation of probable consequences of that behaviour), subjective norm (i.e. perception of others' approval of the behaviour) and perceived behavioural control (PBC; i.e. perception of control based on perception of skills and external obstacles/facilitators). Each of these constructs is based on underlying beliefs. Several extensions of the TPB have been proposed [36]; for example, personal norm (personal moral evaluation of the behaviour), descriptive norm (perception of others' performance of the behaviour [37]), habit [38] and anticipated regret (the regret one experiences when prospectively imagining having—or not having—performed a behaviour [39,40]).

Five studies [24,25,27,31,33] tested expectancy models (e.g. [41]), which propose that behaviour is determined by expectations people have of the behaviours' consequences. Two studies [24,25] assessed how often participants used ecstasy for particular reasons (e.g. ‘in the past year, how often have you used ecstasy to help you to let go of inhibitions?’), implying germane expectations (such as ‘taking ecstasy helps me let go of inhibitions’). Three studies [27,31,33] used more traditional measures (e.g. ‘I have experienced/would expect that ecstasy makes it easier to communicate’). Note that these expectations are viewed as underlying beliefs in the TPB, particularly in relation to the structure of attitudes [12,35].

In both the TPB and outcome expectancy models, higher-level constructs, such as attitudes, are based on lower-level beliefs. Most studies tested models involving this hierarchical cognitive structure. For the purposes of this review, higher-level constructs are referred to as ‘compound constructs’ and lower-order cognitions as ‘expectancies’ (e.g. beliefs about probable consequences of ecstasy use). Details of the particular theoretical models explored in the original studies are not provided here.

Only bivariate analyses were synthesized because multivariate analyses were incommensurable over studies as they tested different models. [In order to resolve this by conducting uniform regression analyses, all authors were asked to provide matrices of covariance. However, due to lost data sets, missing data and non-response, not enough data could be retrieved to render this feasible. The authors are grateful to M. Conner and T. ter Bogt, who did supply additional data.] It is worth noting none the less that in the two TPB-based studiesin which ecstasy use was regressed onto compound constructs, the average (weighed) *R*^2^ was 0.51 [26b,^28^], and in the five TPB-based studies in which intention to use was regressed onto compound constructs the average *R*^2^ was 0.67 [26a,26b,^28^,^30^,^32^]. The average *R*^2^ for the three expectancy studies in which ecstasy use was regressed onto expectancies was 0.35 [24,27,31], and in the expectancy study where intention to use was regressed onto expectancies was 0.64 [25]. In three prospective studies ecstasy use was found to be related strongly to prior intention to use with an average (weighed) *r* of 0.71 [26b,^28^,^30^].

Table 2 shows the strength of association between predictor variables and measures of ecstasy use and intention to use. The strongest predictor of intention and use was the TPB-specified attitude measure. Attitudes are thought to be based on more specific expectancies [12,35] and these are shown separately in Table 2, grouped into ‘positive’ and ‘negative’ expectancies. In addition to these perceived advantages and disadvantages of ecstasy use, normative measures have been used, especially subjective and descriptive norms, as well as perceived behavioural control over ecstasy use. Variables proposed as extensions to the TPB have been categorized as ‘miscellaneous’.

**Table 2:** Effect sizes of predictors of ecstasy use and intention to use.

		Association with behaviour		Association with intention	
			
Variable type	Compound or expectancy	k	r^+^	k	r^+^
Attitude	Attitude [C]	5	0.53 (L)	5	0.63 (L)
Positive expectancies	Social function [C]	4	0.09	2	0.30 (M)
	Produces a positive mood state	3	0.12 (S)	2	0.28 (M)
	Mood function [C]	4	0.20 (S)	1	0.41 (L)
	Helps lose weight	2	0.09	1	0.08
	Enhances sex	3	0.11 (S)		
	Helps stay awake	3	0.08		
	Helps keep fit	1	0.14 (S)	1	0.22 (S)
	Enhances insight/openness	2	0.18 (S)		
	Helps to relax/helps coping	2	0.08		
	Produces excitement			1	0.31 (M)
	Produces intoxication	1	0.19 (S)		
	Eases after-effects	1	0.15 (S)		
	Improves other drugs' effects	1	0.11 (S)		
	Is conforming to peers	1	0.05		
	Helps work	1	0.05		
Negative expectancies	Short-term negative effects [C]	4	−0.47 (L)		
	Produces mood swings	1	−0.34 (M)	2	−0.31 (M)
	Leads on to more frequent use	1	−0.30 (M)	2	−0.39 (L)
	Produces mental side-effects	1	−0.24 (M)	2	−0.40 (L)
	Leads on to ‘worse’ drugs	1	−0.23 (S)	2	−0.31 (M)
	Produces physical side-effects	1	−0.18 (S)	2	−0.39 (L)
	Makes one unhealthy	1	−0.18 (S)	2	−0.30 (M)
	Produces depression	1	−0.18 (S)	2	−0.28 (M)
	Produces lethargy	1	−0.05	2	−0.20 (S)
	Produces addiction	1	−0.32 (M)	1	−0.12 (S)
	Leads to death	1	−0.30 (M)	1	−0.32 (M)
	Produces paranoia	1	−0.27 (M)	1	−0.26 (M)
	Makes one feel run down	1	−0.17 (S)	1	−0.25 (M)
Subjective injunctive norm	Subjective norms (approval) [C]	5	0.39 (L)	5	0.52 (L)
	Best friends' approval	1	0.36 (M)	1	0.25 (M)
	Partners approval	1	0.15 (S)	1	0.30 (M)
	Parents' approval	1	0.10 (S)	1	0.01
	Health experts' approval	1	0.09	1	0.00
	Other ecstasy users' approval	1	0.07	1	0.08
Subjective descriptive norm	Subjective norms (descriptive) [C]	1	0.52 (L)	1	0.63 (L)
	Perceived peer use	2	0.39 (L)	1	0.43 (L)
	Perceived use in close relatives	2	0.21 (S)		
	Perceived best friend/partner use	1	0.37 (L)		
Perceived behavioural control	PBC [C]	3	0.33 (M)	3	0.52 (L)
	PBC (over obtaining ecstasy)	2	0.20 (M)	2	0.25 (M)
	PBC (over taking ecstasy)	2	0.01	2	−0.03
	Being with friends who use	1	0.45 (L)	2	0.62 (L)
	Going out dancing	1	0.41 (L)	2	0.67 (L)
	Being offered ecstasy	1	0.40 (L)	2	0.59 (L)
	Ecstasy being available	1	0.40 (L)	2	0.57 (L)
	Cheap ecstasy	1	0.19 (M)	2	0.54 (L)
	Having alcohol	1	−0.02	2	0.10 (S)
	PBC (over *not* taking ecstasy)	1	−0.06	1	0.07
	Needing to lose weight	1	−0.05	1	0.00
	Needing to exercise	1	−0.01	1	0.05
Miscellaneous	Habit [C]	2	0.45 (L)	2	0.46 (L)
	Moral norm [C]	2	−0.28 (M)	2	−0.31 (M)
	Denial of negative consequences	1	0.17 (S)	1	0.18 (S)
	Anticipated regret	1	−0.11 (S)	1	−0.22 (S)

[C] = compound construct, *k* = no. of samples, *r*+ = weighed average correlation, (S) = small, (M) = medium, (L) = large effect size magnitude according to Cohen [42]. PBC: perceived behavioural control.

Table 2 is based on the Pearson correlations reported in five studies [24,25,28,30,32] and correlation matrices received from the authors of two publications [26,31]. One study [27] reported *t*-values, which were converted into effect size correlations using *r* = √[*t*^*2*^/(*t*^*2*^ + df)]. The results from two studies [29,33] could not be converted and will be provided later. Only associations found in at least two samples, of magnitudes corresponding to a medium (Cohen's *d* = 0.5 [42], *r* = 0.24) or large effect size (*d* = 0.8 and *r* = 0.37), are considered.

All significant associations were in the expected direction and some large effect sizes were observed, suggesting that both the theory of planned behaviour and the expectancy approach can help to explain ecstasy use and intentions to use. In the TPB studies, intention and behaviour are associated most strongly with TPB's attitude, with a large effect size (*r*+ = 0.53 with behaviour, *r*+ = 0.63 with intention). Specifically, this compound construct appears to be associated mainly with positive outcomes regarding mood control and social facilitation, and negative expectations regarding short-term negative effects, escalating use and physical and mental side effects.

Normative influences are also important covariates of use and intention to use with stronger associations observed for descriptive (*r*+ = 0.52 with behaviour, *r*+ = 0.63 with intention) than injunctive norms (*r*+ = 0.39 with behaviour, *r*+ = 0.52 with intention). The strongest effect sizes for expectancies underpinning these compound constructs were observed for perceived ecstasy use by peers and approval of use by one's best friend and partner. The results also indicate that whether parents, health experts and ‘other ecstasy users’ approve is inconsequential.

Perceived behavioural control was related to use with a medium (near large) effect size (*r*+ = 0.33) and also related strongly to intention (*r*+ = 0.52). Underlying beliefs showed a medium effect regarding control over obtaining ecstasy and large effects of control in relation to being with friends who use, going out dancing, being offered ecstasy and ecstasy being available. Two proposed extensions to TPB appear relevant: habit, with a large effect size (*r*+ = 0.45 with behaviour, *r*+ = 0.46 with intention) and moral norm, with a medium effect size (*r*+ = −0.28 with behaviour, *r*+ = −0.31 with intention).

Two studies reported results using statistics that could not be transformed to an effect size measure. One study [29] conducted a polynomial logistic regression predicting user group (six levels: rejectors, at-risk non-users, ex-users, and light, moderate and heavy users; for details, see [29]), testing whether a number of variables contributed significantly to model fit when predicting user group. Another study [33] conducted *t*-tests on beliefs (also predicting user group, with two levels: non-user and user), but did not report *t*-values, exact *P*-values, or variance information. The variables tested in these studies are shown in Table 3. Effect sizes were estimated (conservatively) on the basis of sample size and significance.

**Table 3:** Significant and non-significant predictors of user group (user group had six levels in study [29] and two levels in study [33]).

Variable	Study	Significance	Association	Effect size
Perceived use by friends	[29]	< 0.001	Positive	M
Anticipated regret (‘use would induce guilt’)	[29]	< 0.001	Negative	M
Is hard to resist	[29]	< 0.001	Positive	M
Moral norm (‘ecstasy use is immoral’)	[29]	< 0.05	Negative	S
Perceived availability of ecstasy	[29]	< 0.05	Positive	S
Is bad for one's physical health	[29]	None	–	–
Is bad for one's mental health	[29]	None	–	–
Subjective norm (approval)	[29]	None	–	–
Harmful long-term physical effects	[33]	< 0.01	Negative	M
Risk associated with using regularly	[33]	< 0.01	Negative	M
Risk association with using once or twice	[33]	< 0.05	Negative	M
Harmful long-term psychological effects	[33]	< 0.05	Negative	M
Harmful short-term physical effects	[33]	None	–	–
Harmful short-term psychological effects	[33]	None	–	–
Positive physical effects	[33]	None	–	–
Positive psychological effects	[33]	None	–	–

S = small, M = medium, effect size magnitude according to Cohen [42].

These additional results confirm the relevance of descriptive norm, negative expectancies (particularly long-term effects) and perceived control, and add anticipated regret, with at least a medium effect size, to the list.

## DISCUSSION

Synthesis of the included studies shows the main predictors of intention to use and actual ecstasy use to be attitude (specifically positive outcomes regarding mood control and social facilitation and negative outcomes regarding escalating use and physical and mental side-effects); subjective and descriptive norms regarding one's friends, partner and peers; perceived control regarding obtaining ecstasy and control in relation to being with friends who use, going out dancing, being offered ecstasy and ecstasy being available; and habit, moral norm and anticipated regret. In addition to lending support to both the TPB and expectancy models, these findings show that some expectancies underlying attitude are irrelevant (e.g. ecstasy enhances sex), as are norms relating to some social referents (e.g. parents), and perceived control regarding some ecstasy-related behaviours (e.g. taking ecstasy).

As it is now clear which determinants best predict intention to use and ecstasy use according to the research so far, these determinants seem advisable intervention targets. However, not all determinants are equally easy to modify. As attitude encompasses several specific expectancies, it may be easier to target these more concrete expectancies than the abstract attitude construct. Also, not all expectancies are influenced equally easily. Because most users have experienced positive mood shifts, it may be difficult to develop persuasive messages that undermine this expectancy among users [43]. Negative variables are also associated strongly, and changed more easily. However, simply presenting information about negative outcomes (‘fear appeals’) has been shown not to work, or even work inversely, unless a number of critical conditions are met, such as efficacy enhancement (see [6,44]; also see [45]). Similarly, it may be difficult to change a subjective norm if it reflects reality. As ecstasy use is social [46] and most users take ecstasy at a dance event [47] where ecstasy use is high (in the Netherlands, about two-thirds of the visitors use ecstasy; [48]), it might be hard to reduce descriptive norms, especially if participants' friends use ecstasy. Similarly, it may be challenging to establish a disapproving norm (see [49]).

Similarly, it may be difficult to reduce perceived control over obtaining ecstasy among users, as they probably have repeatedly performed this behaviour successfully. However, the large effects of specific control beliefs suggest that users who wish to stop may well be aided by adopting a stimulus control strategy avoiding the social contexts of use. In addition, enhancing refusal skills would influence perceived behavioural control, while also diminishing the effect of undesirable subjective norms (by decreasing users' motivation to comply, see [35]). Thus, among those with intentions not to use, refusal skills training and stimulus control are recommended [50]. Finally, habit and moral norm are useful predictors but may be difficult to change ([8], but also see [51]). Although anticipated regret has a smaller association with use and intention to use, it can be changed more easily (e.g. [39]). A list of methods and strategies to change these determinants, and the theoretical parameters prerequisite to success, is provided in chapter 7 of Bartholemew *et al*. [8].

This review is limited mainly by the fact that only bivariate associations could be synthesized and by two consequences of the paucity of research into ecstasy use thus far. First, the small number of studies into determinants of ecstasy use limit the robustness of the current findings. Second, many theories and models have not yet been studied and are therefore not included in this review. Only social cognitive theories have been tested; no studies have investigated, for example, the predictive value of implicit cognitions. However, recent research implies that implicit processes may be changeable [52]. Moreover, within social cognitive research, recently developed constructs such as consideration of future consequences [53] have not yet been applied to ecstasy use, but may aid in intervention development.

Regarding the theories that have been studied, in order to gain a more comprehensive understanding of motives for ecstasy use future research should combine the two theoretical perspectives studied thus far, so that relative overlap can be determined. It would be interesting to see whether, and to what degree, particular expectancies account for the predictive utility of the TPB attitude measures. Also, the list of expectancies that has been studied so far may omit a number of consequences (such as ‘suicide Tuesday’, a term for a period following use when low serotonin levels can induce depressive feelings).

Another important gap in the literature concerns behaviours other than ‘using ecstasy’. Not only can the determinants of trying ecstasy out, starting use, ceasing use and maintaining cessation differ from the determinants of using ecstasy [12], little is known about the determinants of harm reduction practices, such as having one's ecstasy tested by a test service, ensuring sufficient hydration and maintaining a low body temperature (although studies such as [54] are a step in the right direction). Intervening to promote these behaviours could prove to be more beneficial to the health of party visitors, given the difficulty of intervening on most variables determining ecstasy use.

In conclusion, this review suggests that there is sufficient evidence to guide intervention development so that evidence-based practice is established. These interventions could then be evaluated to test the utility of particular theoretical frameworks. The priorities for interventions should be negative expectancies, perceived behavioural control and anticipated regret. Tailored interventions can offer refusal skills training and strategies to avoid risky situations to participants not intending to use ecstasy.

## Appendix I

**Table:** Search terms used in PsycINFO (equivalent terms used in corresponding fields in MedLine and ERIC) [query in words, as corresponding to ‘concepts’ column, in brackets].

No.	Concept	Operationalization	Fields
1	Language	(English) or (Dutch)	Language
2	Publication type	(journal*) or (peer-reviewed-journal)	Publication type
3	Publication date	> 1980	Publication year
4	Ecstasy	(clubdrug*) or (club near drug*) or (dance near drug*) or (dancedrug*) or (party near drug*) or (partydrug*) or (xtc) or (mdma) or (methylenedioxymethamphetamine) or (‘3,4-methylenedioxymethamphetamine’) or (ecstasy)	Title, abstract, keywords
5	Theoretical	(theor*) or (attitud*) or (motivat* near functio*) or (mode*) or (norm*) or (perceived near control) or (pbc) or ('social cognitive’) or (self adj efficacy) or (stages near change) or (perceived adj (harm or risk or functions)) or (functional) or (outcome adj (expectancies or expectations)) or (sct) or (tpb) or (patter*) or (psychosoc*) or (health adj belief adj model) or (hbm)	Title, abstract, keywords
6	Determinants	(determin*) or (facto*) or (variabl*) or (parameter*) or (reason*) or (caus*) or (motiv*) or (incentive*) or (correlat*) or (antecedent*) or (character*)	Title, abstract, keywords
7	Initiation	(start*) or (commenc*) or (originat*) or (onset) or (initiat*) or (instigat*) or ((use) not (user)) or (using) or (usage) or (establish*)	Title, abstract, keywords
8	Maintenance	(maint*) or (sustain*) or (continu*) or (uphold*) or (persist*) or (further*) or (prolong*)	Title, abstract, keywords
9	Cessation	(end*) or (stop*) or (discontinu*) or (terminat*) or (ceas*) or (cessat*) or (abstain*) or (abstin*) or (quit*) or (remiss*) or (resolut*) or (recover*)	Title, abstract, keywords
10	Harm reduction	(harm or risk or damage or casualt*) and (reduc* or manag* or limit* or minimi*)	Title, abstract, keywords
11	Excluded	((treatment not (‘not in treatment’ or ‘non-treatment’ or ‘non- treatment’ or ‘no treatment’)) or rat or rats or mouse or mice or animal or monkey* or pigeon* or spectro* or cardio* or seroton* or dopamin* or neurotransm* or receptor* or psychiatr* or psychopath* or cell* or diagnos*)	Anywhere
12	Inclusion	#1 and #2 and #3 [Language and Publication Type and Publication Date]	–
13	Behaviour	#7 or #8 or #9 or #10 [Initiation or Maintenance or Cessation or Harm reduction]	–
14	Empirical*	#6 near #13 [Determinants near Behaviour]	–
15	Final query*	#12 and #4 near (#5 or #14) not #11 [Inclusion and Ecstasy near (Theoretical or Empirical) not Excluded]	–

When executed, the query consisted of one command; therefore the use of the ‘near’-operator was valid here.

## Appendix II

**Table:** Search procedure, number of resulting hits, and results of each step.

Step	Activity	Number of resulting publications
1	Input of query at 20 August 2007 in PsycINFO (162), MedLine (194) and ERIC (11)	367
2	Removal of duplicate records (83)	284
3	Removal of records about publications that (entries were removed in this order):	
3.1	studied biological variables (e.g. sequelae of ecstasy use; 75)	209
3.2	did not study ecstasy use or a related behaviour (such as trying out ecstasy, ceasing use, changing use patterns, or applying harm reduction practices; 32)	177
3.3	studied variables that cannot be changed using a health promotion intervention (e.g. sex, ethnicity or religion; 68)	109
3.4	studied ecstasy use as an independent variable in a multivariate or longitudinal analysis (22)	87
3.5	did not employ quantitative methods (e.g. qualitative studies; 30)	57
3.6	did not study behaviour or cognitions (25)	32
3.7	studied a ‘non-normal’ subpopulation or gathering data from samples inseparably encompassing these subpopulations (e.g. dependent participants, patients or delinquents)*, studying generic drug categories (e.g. ‘hard drugs’)†, or not explicitly stating which drugs were studied (14)	18
3.8	were not published in a peer-reviewed journal (3)	15

* inclusion of these subpopulations would restrict generalization of the results to the target population of the current study (i.e. the average adolescent; see [12,55]).

† this demand of drug specificity is necessary because previous research has shown that beliefs about drugs can vary between different drugs [56], rendering aggregation questionable.
